# Variations in the course of the maxillary artery in Japanese adults

**DOI:** 10.1007/s12565-012-0146-x

**Published:** 2012-07-22

**Authors:** Shingo Maeda, Yukio Aizawa, Katsuji Kumaki, Ikuo Kageyama

**Affiliations:** Department of Anatomy, School of Life Dentistry at Niigata, Nippon Dental University, 1-8 Hamaura-cho, Chuo-ku, Niigata, 951-8580 Japan

**Keywords:** Maxillary artery, Inferior alveolar artery, Middle meningeal artery, Posterior deep temporal artery, Lateral pterygoid muscle

## Abstract

Many authors have studied variation in the maxillary artery but there have been inconsistencies between reported observations. The present research aimed to examine the courses and branching patterns of the trunk and branches of the maxillary artery in a large sample of Japanese adult cadavers. The course of the maxillary artery should be reclassified into seven groups as a clear relationship was found between the origin of the middle meningeal artery and the course of the maxillary artery. This indicates that conventional theory about the formation of the maxillary artery, which was considered to be a direct derivative of the stapedial artery, might be inaccurate. Many variations in the origin of the inferior alveolar artery were found. Notably, the inferior alveolar artery origin from the external carotid artery and a double origin of the inferior alveolar artery was also observed. Thus, the maxillary artery might be derived from a combination of both the external carotid and stapedial arteries.

## Introduction

Variations in the maxillary artery have been studied by many investigators (Thomson 1891; Adachi 1928; Fujita 1932; Kijima 1932; Lurje 1947; Lasker et al. 1951; Takarada 1958; Krizan 1960; Ikakura 1961; Skopakoff 1968; Czerwinski 1981; Iwamoto et al. 1981; Sashi 1989; Tsuda 1991; Otake et al. 2011). The frequencies of the maxillary artery running medially to the lateral pterygoid have been reported as follows: 7.3 % in Mongoloid (Japanese), 38.0 % in Caucasoid (Table 1). The type of maxillary artery that runs medially to the mandibular nerve is rare in many populations. Also, it is important to know that the branching order and formation of the maxillary artery are variable.

**Table 1:** Frequencies of different types of maxillary artery

Reference	Cases	Lateral type (%)	Medial type (%)
Mongoloids (Japanese)			
Adachi (1928)	331	310 (93.7)	21 (6.3)
Fujita (1932)	119	107 (89.9)	12 (10.1)
Kijima (1932)	20	19 (95.0)	1 (5.0)
Takarada (1958)	120	109 (90.8)	11 (9.2)
Ikakura (1961)	160	145 (90.6)	15 (9.4)
Iwamoto et al. (1981)	158	147 (93.0)	11 (7.0)
Sashi (1989)	100	93 (93.0)	7 (7.0)
Tsuda (1991)	339	317 (93.5)	22 (6.5)
Otake et al. (2011)	28	27 (96.4)	1 (3.6)
Total	1,375	1,274 (92.7)	101 (7.3)
Caucasoids			
Thomson (1891)	447	243 (54.4)	200 (44.7)
Lurje (1947)	152	103 (67.8)	49 (32.2)
Lasker et al. (1951)	147	80 (54.4)	67 (45.6)
Krizan (1960)	200	132 (66.0)	68 (34.0)
Skopakoff (1968)	180	125 (69.4)	55 (30.6)
Czerwinski (1981)	240	158 (65.8)	82 (34.2)
Total	1,366	841 (61.6)	521 (38.0)

The course of the maxillary artery has been classified into three types by Loth (1931) or five types by Fujita (1932) (Table 2). Two other courses of the maxillary artery were reported by Tanaka et al. (2003), Tadokoro et al. (2007) and Fujimura et al. (2009) and, especially in Tanaka’s investigation, the maxillary artery passed through the auriculotemporal nerve. Tadokoro et al. (2007) and Fujimura et al. (2009) reported the maxillary artery passing through the temporal muscle. A new classification is needed for summarizing of the course of the maxillary artery.

**Table 2:** Comparison of classifications of the maxillary artery by previous authors and present study

Type	Loth (1931)	Fujita (1932)	Tanaka et al. (2003)	Tadokoro et al. (2007)	Fujimura et al. (2009)	Present study
Lateral				○	○	Group A
	I	A				Group B
		B				
Intermediate						Group C
Medial	II	C				Group D
		D				Group E
	III	E				Group F
			○			Group G

This research aimed to examine the courses and branching patterns of the trunk and branches of the maxillary artery in a large sample of Japanese adult cadavers to determine if a new classification for the course of the maxillary artery should be established and whether the branching patterns of the maxillary artery, especially the middle meningeal, the inferior alveolar and the posterior deep temporal arteries, need to be reconsidered.

## Materials and methods

The topographical relationships among the maxillary, the middle meningeal, the inferior alveolar and the posterior deep temporal arteries were studied in a total of 208 sides of 104 adult Japanese cadavers (54 males, 50 females) available for student dissection at the Nippon Dental University School of Life Dentistry at Niigata.

After the coronoid process was transected, the temporalis was reflected superiorly to expose the infratemporal fossa. The relationship between the lateral pterygoid and the maxillary artery and the courses of the superficial branches were recorded, and the lateral pterygoid subsequently removed. The maxillary artery, its branches, and the surrounding nerves were recorded in detail using digital photographs (Nikon D40x and Tamron SP AF 60 mm F/2 Di2 MACRO 1:1).

## Results

The course of the maxillary artery was classified into the following three types: lateral, intermediate and medial type.

**Fig. 1:**
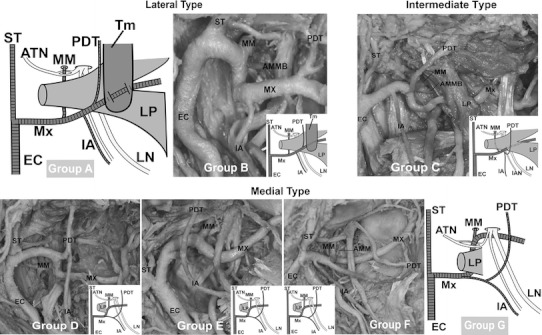
Our classification of the maxillary artery. Groups A [reported by Tadokoro et al. (2007) and Fujimura et al. (2009)] and G [reported by Tanaka et al. (2003)] were not observed in this study

**Fig. 2:**
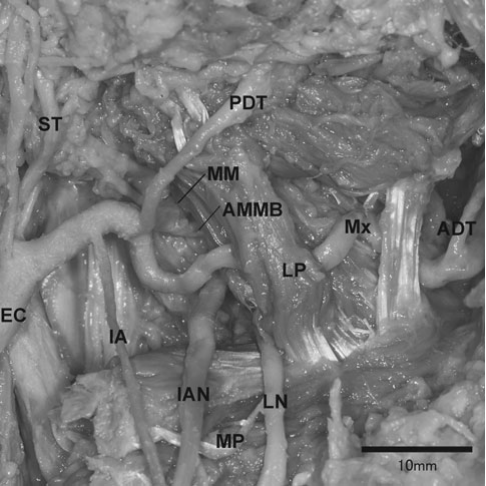
The intermediate type

**Fig. 3:**
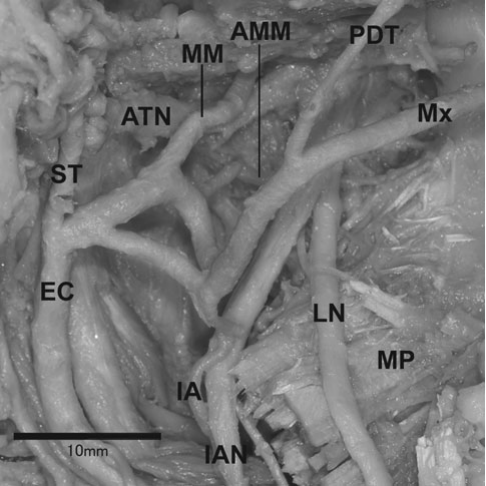
Complete loop of the stems of the maxillary artery

The lateral type of the maxillary artery running laterally to the lateral pterygoid was classified as either group A or B. The intermediate type of the maxillary artery running through the lateral pterygoid was classified as group C. The medial type of the maxillary artery running medially to the lateral pterygoid was classified into four groups: D, E, F and G (Fig. 1). With regards to the course of the maxillary artery, subjects exhibiting the classification of group B were observed in 188 sides (90.4 %) (101 males: 93.5 %, 87 females: 87.0 %). The number and the frequency of the other types of the maxillary artery are listed in Table 3. Eighty-nine cadavers (85.6 %) (48/54 males: 88.9 %, 41/50 females: 82.0 %) were observed bilaterally in group B (Table 4). Four cadavers (3.9 %) (1/54 male: 1.8 %, 3/50 females: 6.0 %) were observed bilaterally as the medial type where the course of the maxillary artery runs medially to the lateral pterygoid in groups D, E and F (Table 4). The intermediate type of the maxillary artery was found in three cases (1.4 %) (3/100 females: 3.0 %) in Group C (Fig. 2; Table 3). Although groups A and G were not observed in this study, group A and G were already classified by Tanaka et al. (2003) and Tadokoro et al. (2007). In one particular case, the maxillary artery bifurcated into a superficial trunk and a deep trunk in the proximal part. In this case, the middle meningeal and the accessory middle meningeal arteries arose from the deep trunk and the inferior alveolar artery originated from the superficial trunk respectively. Both trunks reunited to form a complete loop near the point of the maxillary artery crossing the anterior margin of the lateral pterygoid (Fig. 3).

**Table 3:** Course of the trunk of the maxillary artery

Type	Group	Males			Females			Males and females		
		Right	Left	Total	Right	Left	Total	Right	Left	Total
Lateral	A	0	0	0	0	0	0	0	0	0
	B	50	51	101	43	44	87	93	95	188
		(92.6 %)	(94.4 %)	(93.5 %)	(86.0 %)	(88.0 %)	(87.0 %)	(89.4 %)	(91.3 %)	(90.4 %)
Intermediate	C	0	0	0	2	1	3	2	1	3
					(4.0 %)	(2.0 %)	(3.0 %)	(1.9 %)	(1.0 %)	(1.4 %)
Medial	D	3	3	6	5	1	6	8	4	12
		(5.6 %)	(5.6 %)	(5.6 %)	(10.0 %)	(2.0 %)	(6.0 %)	(7.7 %)	(3.8 %)	(5.8 %)
	E	1	0	1	0	3	3	1	3	4
		(1.8 %)		(0.9 %)		(6.0 %)	(3.0 %)	(1.0 %)	(2.9 %)	(1.9 %)
	F	0	0	0	0	1	1	0	1	1
						(2.0 %)	(1.0 %)		(1.0 %)	(0.5 %)
	G	0	0	0	0	0	0	0	0	0
*n* = side		*n* = 54	*n* = 54	*n* = 108	*n* = 50	*n* = 50	*n* = 100	*n* = 104	*n* = 104	*n* = 208

**Table 4:** Combination of the course of the maxillary artery

Males	Left							Total
Cadavers (*n* = 54)	Group A	Group B	Group C	Group D	Group E	Group F	Group G	
Right									
Group A	0	0	0	0	0	0	0	0
Group B	0	48 (88.9 %)	0	2 (3.7 %)	0	0	0	50 (92.6 %)
Group C	0	0	0	0	0	0	0	0
Group D	0	3 (5.6 %)	0	0	0	0	0	3 (5.6 %)
Group E	0	0	0	1 (1.8 %)	0	0	0	1 (1.8 %)
Group F	0	0	0	0	0	0	0	0
Group G	0	0	0	0	0	0	0	0
Total	0	51 (94.4 %)	0	3 (5.6 %)	0	0	0	54 (100 %)

Females	Left							Total
Cadavers (*n* = 50)	Group A	Group B	Group C	Group D	Group E	Group F	Group G	
Right									
Group A	0	0	0	0	0	0	0	0
Group B	0	41 (82.0 %)	0	0	2 (4.0 %)	0	0	43 (86.0 %)
Group C	0	1 (2.0 %)	1 (2.0 %)	0	0	0	0	2 (4.0 %)
Group D	0	2 (4.0 %)	0	1 (2.0 %)	1 (2.0 %)	1 (2.0 %)	0	5 (10.0 %)
Group E	0	0	0	0	0	0	0	0
Group F	0	0	0	0	0	0	0	0
Group G	0	0	0	0	0	0	0	0
Total	0	44 (88.0 %)	1 (2.0 %)	1 (2.0 %)	3 (6.0 %)	1 (2.0 %)	0	50 (100 %)

Males and females	Left							Total
Cadavers (*n* = 104)	Group A	Group B	Group C	Group D	Group E	Group F	Group G	
Right									
Group A	0	0	0	0	0	0	0	0
Group B	0	89 (85.6 %)	0	2 (1.9 %)	2 (1.9 %)	0	0	93 (89.4 %)
Group C	0	1 (0.9 %)	1 (0.9 %)	0	0	0	0	2 (1.9 %)
Group D	0	5 (4.8 %)	0	1 (0.9 %)	1 (0.9 %)	1 (0.9 %)	0	8 (7.8 %)
Group E	0	0	0	1 (0.9 %)	0	0	0	1 (0.9 %)
Group F	0	0	0	0	0	0	0	0
Group G	0	0	0	0	0	0	0	0
Total	0	95 (91.4 %)	1 (0.9 %)	4 (3.9 %)	3 (2.9 %)	1 (0.9 %)	0	104 (100 %)

### Branching patterns of the main branches of the maxillary artery

**Fig. 4:**
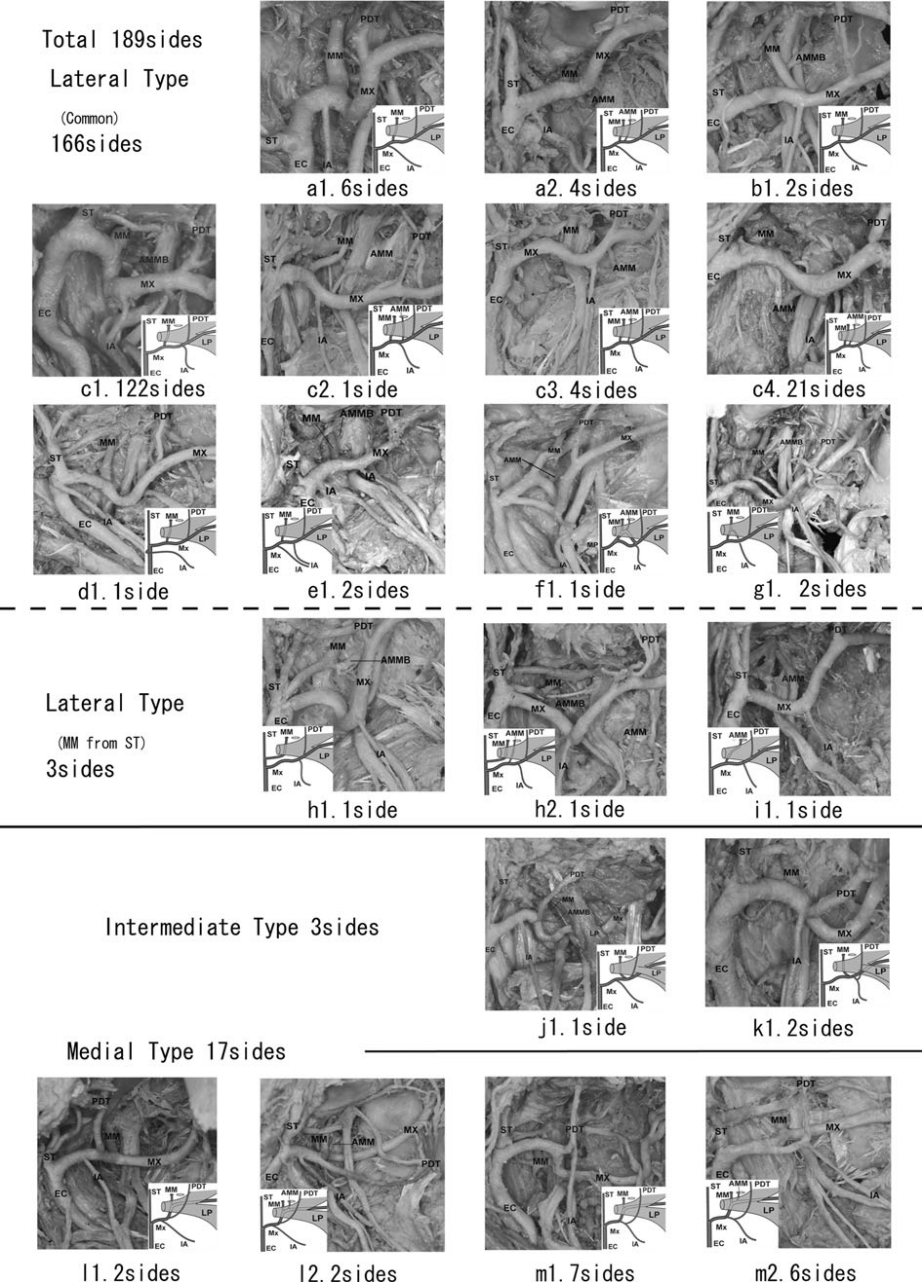
Diagram of 20 sub-patterns of the maxillary artery

**Fig. 5:**
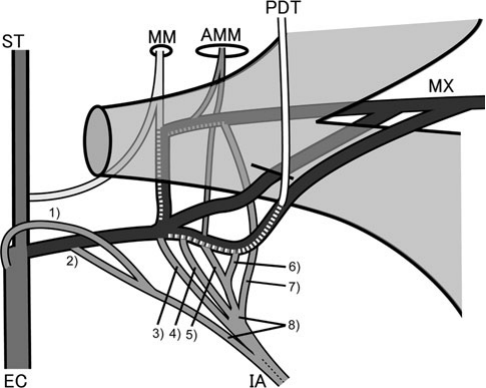
Diagram showing all of the variations in the origin of the inferior alveolar artery (IA). *1* External carotid artery (EC), *2* proximal part of the maxillary artery (Mx), *3* Mx at the same point of the origin of the MM, *4* Mx between the origin of the MM and accessory middle meningeal artery (AMM), *5* Mx at the same point of the origin of the AMM, *6* Mx between the origin of the AMM and the posterior deep temporal artery (PDT), *7* MM, *8* double origins of the inferior alveolar artery (IA)

A total of 189 sides that exhibited branching from the maxillary artery could be confirmed in 208 sides; the branches consisted of the middle meningeal artery, the inferior alveolar artery, and the posterior deep temporal artery. The branching patterns of the main branches of the maxillary artery in 189 sides were classified into 20 sub-patterns (Fig. 4; Table 5). In all cases from a1 through i1 (Fig. 4), the posterior deep temporal artery derived from the maxillary artery after branching from the middle meningeal or the accessory middle meningeal arteries. In cases j1 and k1 (Fig. 4), the middle meningeal artery arose from the superficial temporal artery. In cases j1 and k1 (Fig. 4), the middle meningeal and the posterior deep temporal arteries arose from the same part of the maxillary artery. In cases l1 through m2 (Fig. 4), the posterior deep temporal artery derived from the maxillary artery before the branching of the middle meningeal artery. The summary of all cases regarding the origin of the middle meningeal, the accessory middle meningeal, the inferior alveolar and the posterior deep temporal arteries are shown in Fig. 5.

**Table 5:** Branching order of the main four branches of the maxillary artery.

Sides (*n* = 189)^a^	Group				
	B	C	D	E	F
Lateral (MM − PDT)					
(a) IA − MM − PDT	10				
(b) MM = IA − PDT	2				
(c) MM − IA − PDT	148				
(d) MM − PDT	1				
(e) IA − MM − IA − PDT	2				
(f) (MM − AMM − IA) − PDT	1				
(g) MM + IA − PDT	2				
Total	166				
Lateral (others)					
(h) IA − PDT (MM from ST)	2				
(i) AMM − IA (MM lacking)	1				
Total	3				
Intermediate (MM = PDT)					
(j) IA − MM = PDT		1			
(k) MM = PDT + IA		2			
Total		3			
Medial (PDT − MM)					
(l) IA − PDT − MM			3		1
(m) IA + PDT − MM			9	4	
Total			12	4	1
Group total	169	3	12	4	1

* MM* Middle meningeal artery,* PDT* posterior deep temporal artery,* IA* inferior alveolar artery,* AMM* accessory middle meningeal artery,* ST* superficial temporal artery

^a^−, branch in sequence from mesial to distal; +, common trunk; =, branch arising from the same position

## Discussion

It is known that there are large differences in the frequency of the medial course of the maxillary artery between Caucasoids and Mongoloids (Japanese) (Table 1). The frequency of the medial type in the present study correlates with that of other Japanese authors (Tables 1, 3). However, the frequency of the medial type found in Caucasoids tended to be significantly higher than in Mongoloids (Japanese).

Regarding the course of the maxillary artery, two classification methods have been introduced previously by Loth and Fujita. Fujita’s type D and E were elaborated upon in more detail by Takemura et al. (1983). In this study, group B corresponds to type A and B of Fujita’s method, which were divided from the buccal nerve (Fig. 1; Table 2). The buccal nerve could not be observed in most cases due to alterations made during student dissection course work, therefore group B includes Fujita’s type A and B. This present study reclassification takes into account the fact that the maxillary artery is positioned occasionally between two bundles of the buccal nerve. Two other types of maxillary artery were reported with one passing through the loop of the auriculotemporal nerve at the deepest region (Tanaka et al. 2003), and the other running through the temporalis at its most superficial course (Tadokoro et al. 2007; Fujimura et al. 2009). Three cases (1.4 %) of the maxillary artery passing through the lower head of the lateral pterygoid were observed in this study (Fig. 2). Although similar cases were reported by Adachi (1928), Iwamoto et al. (1981), Takemura et al. (1984), and Fujimura et al. (1991), those authors provided additional classification of the medial type or lateral type. This study shows that the maxillary artery should be classified as “the intermediate type” independently.

In one particularly different case, a divided and reunited maxillary artery was classified tentatively into group B (Fig. 3). Claire et al. (2011) reported a case of the divided and reunited maxillary artery in the infratemporal region. According to Claire’s study, the maxillary artery bifurcated into the deep and superficial branches at the distal part of the divergence of the anterior tympanic artery. The deep and superficial branches reunited to form a complete loop at the infratemporal region. The course of the maxillary artery converged at the anterior margin of the infratemporal fossa in every case. The arterial loop, which proximally reunited between the deep and superficial branches, was observed. In the previous descriptions of divided maxillary arteries, the two branches did not reunite (Lauber 1901; Tadokoro et al. 2008). The middle meningeal artery originated from the deep trunk of the maxillary artery and the inferior alveolar artery arose from the superficial trunk. Therefore, some part of the deep trunk in this case may be equivalent to the trunk of the medial type of the maxillary artery.

Padget (1948) reported the internal maxillary branch (the maxillary artery) of the external carotid artery has now established a connection with the lower division of the stapedial artery at the junction of its maxillary and mandibular branches (the inferior alveolar artery). As described by Tandler (1902), the internal maxillary branch gains the outer side of the mandibular nerve root by the development of an arterial loop around the nerve with subsequent obliteration of the primary medial limb of the loop. As soon as the common trunk of the maxillomandibular division of the stapedial artery becomes surrounded by the auriculotemporal nerve, the part of this trunk that lies above the recently completed anastomosis with the internal maxillary artery (the maxillary artery) becomes recognizable as the stem of the middle meningeal artery. This suggests that the mechanism of the formation of the maxillary artery may not be simple.

The furcation point of the middle meningeal and the posterior deep temporal arteries might be substantially related to the course of the maxillary artery (Figs. 4, 5; Table 5). Conventionally the inferior alveolar artery might be an extension of the middle meningeal artery, therefore the inferior alveolar artery is the third branch of the stapedial artery (Tandler 1902; Padget 1948).

Tandler (1902) and Padget (1948) maintain that the inferior alveolar artery was formed by the third branch of the stapedial artery. The lateral, intermediate and the medial types could not be clarified, namely (Fig. 5, parts 2–6). (1) The inferior alveolar artery arose from the external carotid artery (Fig. 5-1). (2) The inferior alveolar artery originated from the middle meningeal artery (Fig. 5-7). (3) The inferior alveolar artery had a double origin (Fig. 5-8). (4) The middle meningeal artery arose from the superficial temporal artery (Fig. 4-h1, -h2). Therefore, we suggest the inferior alveolar artery arises not only from the extension of the third branch of the stapedial artery but also from other possible arteries.

## Abbreviations

AMM: Accessory middle meningeal artery
AMMB: Accessory middle meningeal branch
ADT: Anterior deep temporal artery
ATN: Auriculotemporal nerve
Bc: Buccinator
EC: External carotid artery
IA: Inferior alveolar artery
IAN: Inferior alveolar nerve
LN: Lingual nerve
LP: Lateral pterygoid
MM: Middle meningeal artery
Mx: Maxillary artery
PDT: Posterior deep temporal artery
ST: Superficial temporal artery
Tm: Temporal muscle
MP: Medial pterygoid
